# Innovative Bacterial Therapies and Genetic Engineering Approaches in Colorectal Cancer: A Review of Emerging Strategies and Clinical Implications

**DOI:** 10.4014/jmb.2408.08026

**Published:** 2024-09-25

**Authors:** Chunxiao Song, Chunwu Zhao

**Affiliations:** 1 Department of Colorectal and Anal Surgery, Weifang People's Hospital, Weifang 261000, P. R. China; 2 Department of Gastrointestinal Surgery, Weifang People's Hospital, Weifang 261000, P. R. China

**Keywords:** Microbiota, bacteriophage therapy, genetic engineering, chromosome instability, CRISPR-Cas9

## Abstract

Colorectal cancer (CRC) is considered a widespread cancer, ranking second in mortality and incidence among cancer patients worldwide. CRC develops from adenoma to carcinoma through the dynamic interplay of genetic and environmental factors. The conventional modes of treatment, including operation, chemotherapy, and irradiation, are associated with significant challenges, such as drug resistance and toxicity, necessitating the exploration of new treatment modalities. These difficulties reveal the necessity of the emergence of new therapeutic approaches. This review mainly emphasizes the bacterial-based therapies that have recently developed like the engineered bacteriophage therapy and bacterial immunotherapy that pale the existing chemotherapy in terms of toxicity but are effective in killing tumor cells. Also, it also investigates various molecular genetic engineering strategies such as CRISPR-Cas9, CRISPR prime editing and gene silencing to achieve better targeting of CRC. Implementing these new approaches into the forefront of CRC treatment may bring better, more effective therapy with fewer side effects on patients’ quality of life.

## Introduction

Colorectal cancer (CRC) is a significant global health problem since it ranks second among the prominent causes of cancer-related mortalities globally [1]. This disease is common in men and women, and the survival rate for five years is comparatively less and estimated to be 65% [1]. When diagnosed, this rate depends on the disease's stage [1]. CRC is a cancer that initiates in the colon or rectum, a section of the large intestine. It starts from a polyp, which is a noncancerous tumor that may grow into a malignant tumor over time [2]. This pathological condition is frequently asymptomatic for many years and is diagnosed in the late stage, which results in a poor prognosis. The manifestation includes frequent changes in bowels, diarrhea, constipation, the urge to defecate, abdominal pain, sometimes passing of bright red blood, either concealed or visible, loss of weight, pain in the abdomen, tiredness, and exhaustion [3]. However, such signs and symptoms are often ignored, and patients do not present themselves to the hospital until the disease is well established, hence the need for regular checkups and screening. This is because the given measures can assist in increasing the success rate of early detection and effective management of the condition [3].

Poor lifestyles, heredity factors, and an undesired diet plan make the populace vulnerable to CRC [4]. The dynamics involved are these layers of regulation of complex genetic mutations and cogent epigenetic modifications that drive the progress of the disease [5]. Knowledge of these risk factors and the molecular processes underlying CRC will provide a platform for developing effective prevention, early screening, and intercession treatment.

The colonic bacterial community is approximately 3 × 10^13^, collectively influencing immunomodulation and immunology [6]. What is more, bacterial structure contains qualities that prevent cancer and trigger inflammation and some features concerning metabolism [6]. Thus, methods of bacterial therapy have been considered in recent years as perspectives in cancer treatments. Some earlier studies proved that specific bacteria like *Streptococcus pyogenes* and *Serratia marcescens* have oncotoxic activity and increase the survival rate of colorectal cancer and sarcoma patients [7]. Recent findings in discovering tumor cell mutations have reverted bacterial therapy into the limelight in managing CRC [8, 9]. However, cancer cells can deploy mechanisms that enable them to avoid detection by the host immune system and become resistant to the existing immunotherapies [10].

Existing remedies for cancer have numerous limitations, strictly the highly detrimental effect of anti-tumor agents on healthy cells taking a toll on one's health [9, 10]. This toxicity often results in side effects and, in most cases, reduces the efficiency of the treatment. To address this problem, the researchers pay attention to bacterial strains like *S. marcescens*, *E. coli*, and *S. pyogenes*, concerning their potential as a CRC treatment rather than traditional therapies in the oncology practice [12]. These bacteria release toxins, mainly endotoxins of low molecular weight, hydrophobic, that can easily penetrate through the tumor cells and thus augment anticancer properties [13, 14].

Although bacterial therapies promise CRC treatment, they have unique challenges and potential risks. These are immune reactions by the host that are sometimes unintended, such as inflammation or systemic infection and the possibility of gene transfer, which could lead to the formation of harmfully genetically modified bacterial strains [15]. Many clinical bacteriophages therapy, CRISPR-Cas9 and bacteria-based therapy for CRC are under trials to test their efficiency and safety. These trials seek to compare potential gains that are therapeutic in nature with the costs of treatment, which may include alteration of favourable gut microbiome or manifestation of other side effects [16, 17]. The aim of this review is to explore the use of bacterial therapies in cancer treatment, especially in CRC. It also discusses the basic principle of using bacteria for cancer treatment and how technological progress including Cas9 has strengthened bacteria’s efficiency in eradicating cancer cells.

## Development of Colorectal Cancer

CRC, diagnosed in different international locations, develops as follows: It begins with adenomas and then extends to the carcinoma stage due to genetic and environmental factors [18]. CRC can arise through molecular patterns, including CIMP, CIN, and MSI [19].

### Chromosomal Instability (CIN) Pathway

**Fig. 1:**
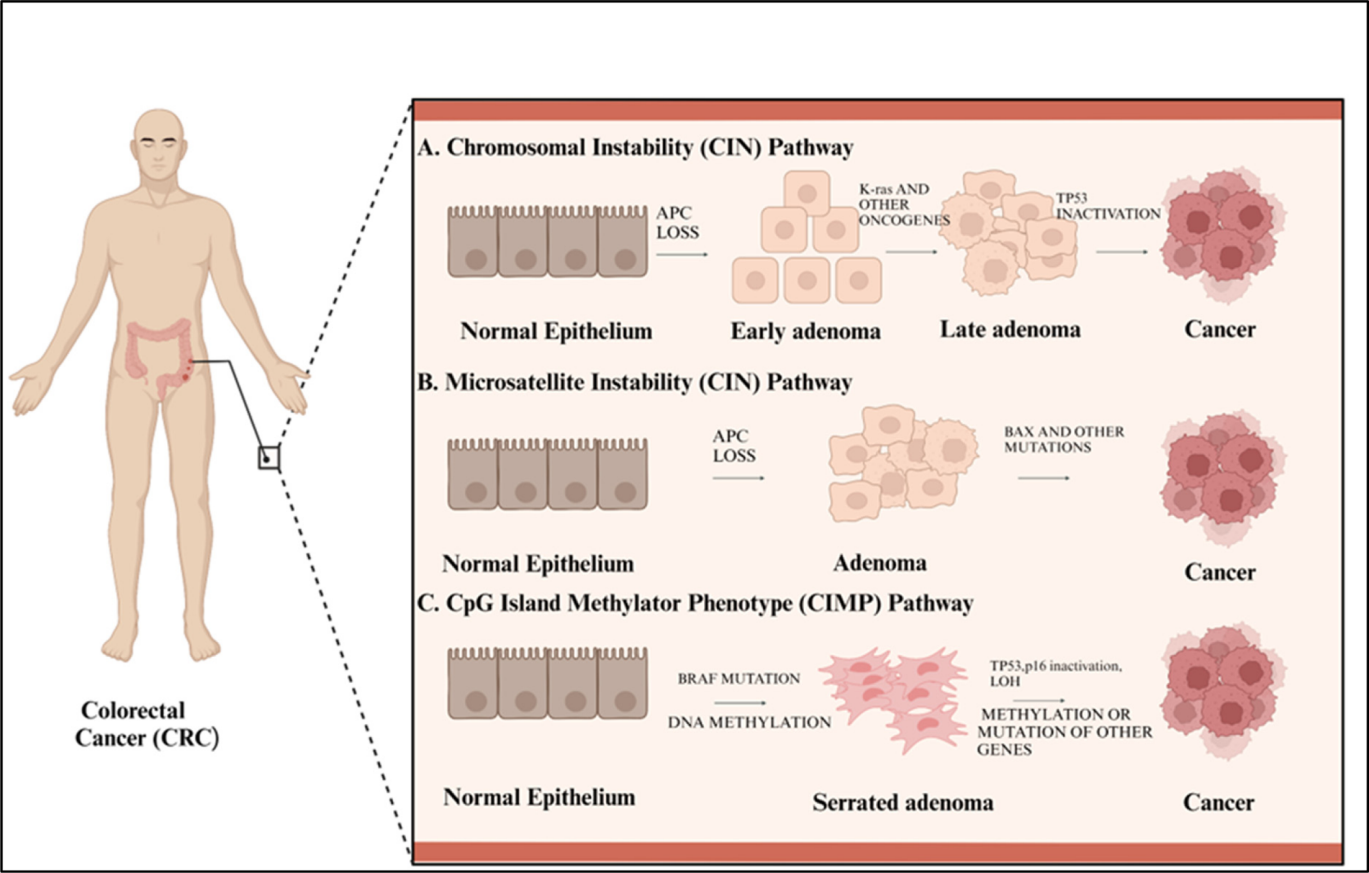
The multi-step progression of colorectal cancer: From normal epithelium to invasive cancer [27].

CIN is the most common event in sporadic colorectal cancer, contributing to 60-75% of the cases. CIN is a situation where a cell gains or loses large parts of the DNA, and this genetic change makes the cell cancerous [20]. This can lead to difficulties in forming a proper structure and organization of chromosomes and the correct number of their cell sets. This instability is often triggered by mutations in genes that code for mitotic checkpoint proteins, such as MAD and BUB that regulate correct chromosome distribution [21]. This genetic mutation leads to the activation of the Wnt signaling pathway, forming adenomatous cells. Activation of Wnt leads to inhibition of beta-catenin degradation, which in normal circumstances is accumulated and translocated to the nucleus, regulating gene expression in the cell and promoting cell growth [22]. However, when APC is not mutated, CTNNB1 mutations are frequently observed, indicating the engagement of this or a similar pathway in primary carcinogenesis [23]. The second main process in the CIN pathway is a mutation of the KRAS gene, leading to the activation of Ras protein. This activation stimulates the division of cells and increases the chances of their death. This activates other signaling pathways, including RAF, MAPK, RALGDS, and PI3K. The third step of the CIN pathway is the accumulation of loss-of-function mutations in the TP53 gene, resulting in the loss of p53 protein function, which leads to uncontrolled cell proliferation (Fig. 1) [24].

### Microsatellite Instability (MSI) Pathway

MSI occurs in approximately 95% of CRC patients with Lynch syndrome [25]. All this information is essential for understanding the disease, its diagnosis, and its treatment. Moreover, MSI is detected in about 15-20% of patients with sporadic CRCs; however, 30-45% of CRCs are associated with Lynch syndrome [26]. MSI defines mutations in repetitive DNA regions within the tumor cells and is divided into two subtypes, MSI-L and MSI-H, based on the number of mutated microsatellites. Also, dwelling on the genetics of CRC, defective DNA mismatch repair (MMR) systems, which include MLH1 and MSH2 cause MSI (Fig. 1). There are hereditary forms of MMR genes that can be mutated by Lynch syndrome. In addition, the loss of MLH1 through epigenetic modifications is a feature of sporadic MSI-H CRC. MSI-H sporadic CRC frequently has mutations in the BRAF, SMAD2, SMAD4, ACVR, and BAX genes [27].

### CpG Island Methylator Phenotype (CIMP) Pathway

The serrated pathway called the CpG Island Methylator Phenotype (CIMP) pathway, is the other described carcinogenic route detected in serrated colorectal lesions. One of these pathways involved epigenetic changes, particularly DNA methylation, where the researchers found that increased promoter methylation was central to modulating Gene expression and the Cancer process [28]. When methylation happens on CpG islands, the transcription of those cytosine-guanine dinucleotide sequences in the promoter region of a gene ceases [29]. There is increased promoter hypermethylation in up to 30% of cases of colorectal cancers, which affects angiogenesis, cell cycle, and apoptosis genes. It is classified into two lists: CRC-CIMP high list and CRC CIMP low list, depending on the levels of CpG island methylation. If three of the five genes tested for have high K27 promoter methylation, then the CRC is category CIMP high; if the number is two or less, then the CRC is CIMP low. Some genes associated with epigenetic silencing by CIMP include P16, MGMT, TIMP3, p14, FHIT, and SLC5A8. The BRAF mutation is an essential node in the serrated pathway. Mutations in both BRAF and KRAS are thought to be mutually exclusive in cases of colorectal cancer despite BRAF's involvement in the KRAS signaling pathway. Uncontrolled cell proliferation and the advancement of cancer are outcomes of BRAF mutation; approximately 90% of CRC cases originate from the serrated pathway, conferring an increased risk of cancer-specific mortality (Fig. 1) [30].

## Epidemiology

Colorectal cancer is the third most prevalent cancer globally, accounting for 10.2% of all cancer diagnoses, with approximately 1.85 million new cases reported annually by the International Agency for Research on Cancer (IARC) [31, 32]. It ranks third among men, after prostate and lung cancers, and second among, after breast cancer. In 2020, CRC resulted in over 930,000 deaths worldwide, with 1.9 million new cases diagnosed in the same year [33]. Geographic variations in incidence and mortality rates are notable, and projections indicate a 73% increase in annual incidence that by 2040, CRC will cause 1.6 million deaths annually and 3.2 million new cases [34, 35].

### Geographical Variations in Colorectal Cancer

The incidence of colorectal cancer (CRC) varies significantly across different regions. Europe has the highest rates, with 28.8-32.1 cases per 100,000 people [36]. Eastern Asia follows with 26.5 cases per 100,000, while North America reports 26.2 cases per 100,000. The incidence rates in Australia and New Zealand are also notably high at 36.7 cases per 100,000. Conversely, South and Central Asia have comparatively lower incidence rates, at 4.9 cases per 100,000. Meanwhile, Africa's rates fall between 6.4 and 9.2 cases per 100,000 [37]. When examining populations aged 0-74, it is interesting to note that the cumulative risk of developing CRC is significantly higher in countries with a very high Human Development Index (HDI) than in countries with a low HDI. On a global scale, the overall risk stands at 2.27%, with men facing a slightly higher risk of 2.75% compared to women's 1.83% [38].

**Fig. 2:**
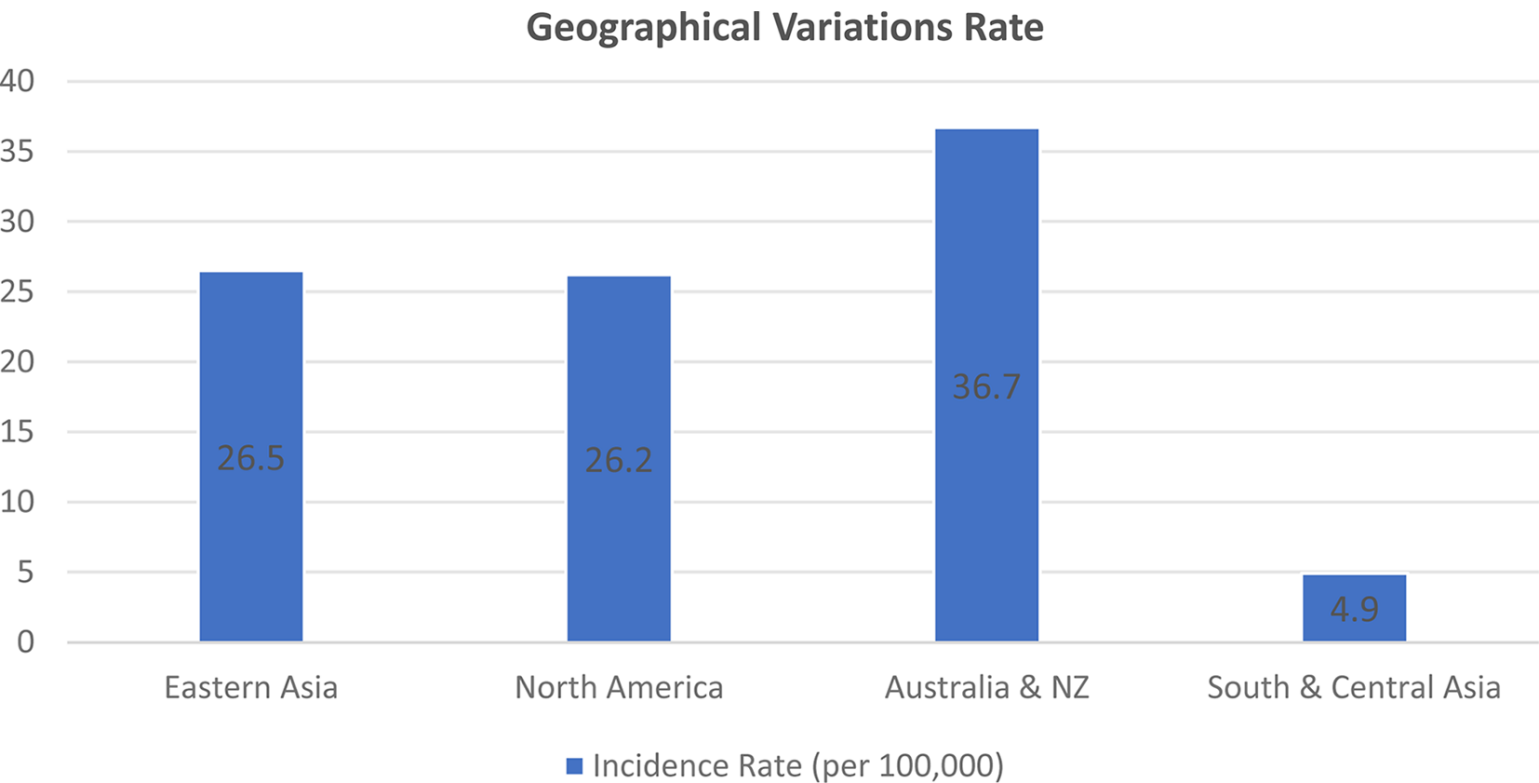
Colorectal cancer incidence rates: A global view with regional insights [39].

Hungary has the highest age-standardized rates, with 51.2 cases per 100,000. South Korea follows closely with 44.5 cases per 100,000, while Slovakia and Norway have rates of 43.8 and 42.9 cases per 100,000, respectively. Although the incidence has recently plateaued or even declined in developed nations, it is rising in some middle -and low-income countries, likely due to adopting Western lifestyles. According to recent studies, about 17% of CRC cases are not detected during routine screenings (Fig. 2) [39].

### Colorectal Cancer Recurrence and Age-Related Risk

**Fig. 3:**
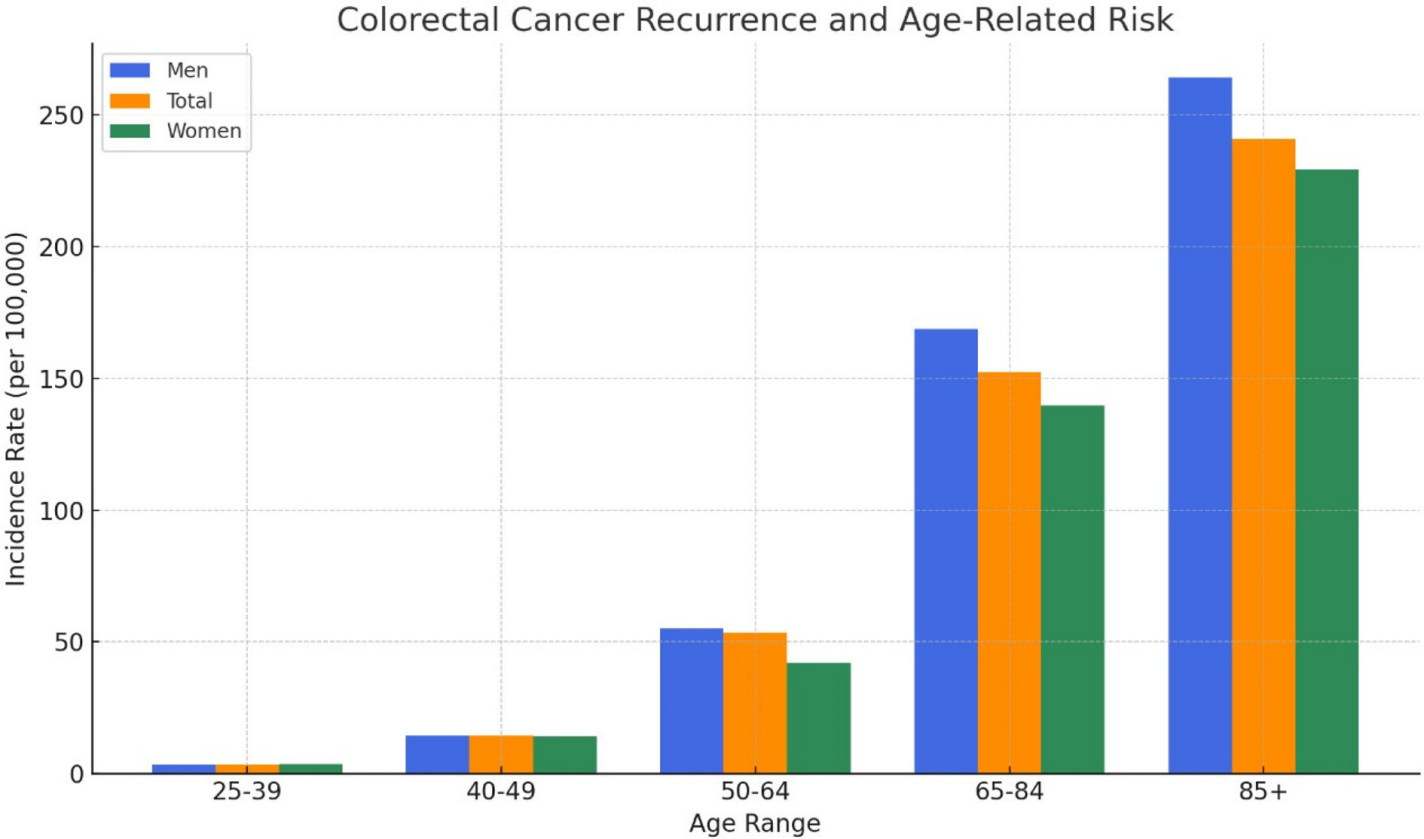
Age-related risk of colorectal cancer recurrence [40].

The incidence of colorectal cancer rises with age, making the elderly a more common target. Compared to men, women have a median age of 72 at diagnosis. Colon cancer rates rise between 25 and 39 years old at a rate of 1.5 to 5.4 per 100,000 people (1.4 to 5.3 for men and 1.6 to 5.5 for women, for women). Within the age bracket of 40-49, the rate rises to 10.3-18.5 per 100,000 (10.2-18.9 for men and 10.4-18.1 for women), 34.4-72.7 per 100,000 (37.4-72.7) for men and 31.2-53.2 for women, 92.6-212.2 per 100,000 (107.7-229.7 for men and 79.2-200.4 for women) between 65-85, and 240.9 per 100,000 (264.0 for men and 229.2 for women) in subjects aged 85 and up. The result is a tenfold increase in the incidence of colon cancer from 50 to 85 years old (Fig. 3) [40].

## Role of Microbiota in Colorectal Cancer

The human microbiome, a complex system of microbial genes, surpasses the human genome in genetic diversity [40]. This microbial consortium plays a crucial role in various body functions, including immunologic reactions and drug metabolism [41]. The current understanding of microbiota has significantly advanced, revealing the profound impact of microbiota on both immunological activity and drug efficacy. For instance, specific bacterial species like *Enterococcus faecalis* and *E. faecium* synthesize tyramine, a neurotransmitter modifier. Others, another study exhibited an elevated utilization of vitamin B5, potentially linked to inflammatory bowel disease pathways. These findings underscore the potential for an improved understanding of microbiota-dependent metabolites in drug therapy, offering hope for future advancements in personalized medicine. This is because most medication-related metabolic changes take place in the gut [42].

### Microbial Dysbiosis and Therapy Resistance

Recent findings show the importance of microbial dynamics in CRC and their potential impact on treatment resistance. An abnormal composition of the gut microbiota also known as microbial dysbiosis may affect the levels of resistance against both bacterial and traditional antibiotic treatments. Understanding how such microbial species or their metabolites influence these resistance mechanisms may help to explain why some interventions work or otherwise. Certain gut microbial species are associated with therapy resistance in CRC. For instance, *Fusobacterium nucleatum* has been associated with chemotherapy resistance because it alters an individual’s immune system to a pro-inflammatory one. Along the similar lines, *E. coli* strains that produce a genotoxin known as colibactin can improve DNA damage and repair mechanisms in tumor cells and can lead to chemo-resistance. Additionally, *Bacteroides fragilis*, produces metabolites able to stimulate tumor cells for enhanced expression of drug efflux proteins thus lowering sensitivity to chemotherapeutic drugs.

Microbes affect therapy resistance through several mechanisms, including drug efflux pumps, DNA repair, and immune avoidance. For example, some bacteria may upregulate the drugs pump or stimulate DNA repair mechanisms and thereby render the chemotherapy ineffective. Further, microbial metabolites can influence the immune system and make cancer cells invisible to the immune system and thus be able to survive chemotherapeutic treatment. Recognition of these interactions and the identification of the exact microorganisms crucial for treatment planning of CRC might help to enhance patients’ outcomes.

### Drug-Microbiota Interactions and Therapeutic Implications

Drug interactions and microbiomes significantly influence the microbiota's composition and balance. Drug-induced toxicity in microbes is evident with anti-diabetics, proton pump inhibitors, and non-steroidal anti-inflammatory drugs, and exhibit toxicity toward microbes [43]. This toxicity can lead to the development of antibiotic resistance, a serious concern in healthcare. Maier *et al*. explored these interactions by exposing 1,197 pharmaceuticals from various therapeutic classes (excluding antibiotics) to 40 distinct bacterial strains. Nearly 30% of the compounds inhibited the growth of at least one bacterial strain, suggesting that non-antibiotic exposure can contribute to antibiotic resistance. Research has identified enzymes that facilitate drug transformation through genetic screens and enzymatic analysis [44].

### Gut Bacterial Metabolism's Impact on Drug Efficacy

Recently, the gut microbiota has been under focus on its metabolism, which may cause disparity in treatment efficacy in patients suffering from the same disease and relying on the same treatment [45]. This also clearly shows the complexity and hazardous task of deciding the treatment approach that best suits a patient. To this end, machine-learning frameworks are used to recognize drug biomarkers that determine drug outcomes and network-mediated approaches. This promising approach results in enhanced individualized cancer treatment and better therapeutic results; consequently, it carries a hopeful perspective for cancer treatment in the future.

Moreover, identifying the toxic bacterial medication by-products allows the prognosis of potential adverse reactions in the patients taking the treatment. The chemical modifications put in place by bacteria can be toxic, neutralizing, or enhancing for the targeted pharmacological agents [46]. For instance, hepatic glucuronidation is a liver metabolic process that affects TNF in response or ROS production and inactivates medications or condenses them to GlcA. On the other hand, the body sends these glucuronides to the intestine and leaves the body. On the other hand, Gut bacteria glucuronidase (GUS) enzymes can activate these compounds by removing the GlcA, which causes acute toxicity in the colon when administered [47]. This study used these techniques: small-molecule structural assay, quantitative metabolomics, metagenomic analysis of the small-molecule structural assay, colonization of mice, and bioinformatics analysis. This led to an approach that was complex and precise at the same time. Applying the medications of different groups with different missions, the authors showed that it was correct to use them to quickly and rapidly identify the MDM enzymes [48].

### The Microbiome's Influence on Conventional Colorectal Cancer Treatments

Standard treatment methods for CRC comprise chemotherapy and radiation treatments for patients directly affecting their gut microbiota, an emerging research subject [49]. Nowadays, chemotherapy has been widely used as the first-line therapy for CRC, but its effectiveness has some shortcomings, leading to the application of adjunct treatment. Current investigations concerning the use of microbiota in therapy have yielded contradictory outcomes. For example, germ-free mice have revealed enhanced irinotecan resistance, and holoxenic mice have described weaker resistance [50]. Drawing such conclusions, one can refer to a somewhat ambiguous influence of gut bacteria within cancer treatment. Thus, the gut microbiota can affect chemotherapeutic agents' utilization and the immune system's capacity to respond to cancer. So the gut microbiome's impact must be considered during the CRC treatment.

Fecal microbiota transplantation (FMT) is a new and highly prospective treatment option or intervention method for different diseases such as autism, inflammatory bowel disease, and diabetes, among others [51]. Moreover, earlier, we have discussed how FMT could also be a safe approach to counter the negative consequences of radiation therapy. Although the concurrent CRT of CRC is well documented, it entails some typical side effects on the bone marrow and gastrointestinal parts, including toxicity [52]. Some bacteria have been evidenced in experiments with rats to mitigate the adverse aftermaths of irradiation, and the gut microbiome might impact the efficacy of radiation therapy, as shown in several preclinical cancer mouse models. For example, *Lactobacillus rhamnosus* displayed the possible ability to preserve intestinal mucosa in irradiated mice. Clinical trials have also indicated that administering a specific type of bacteria probiotics reduces diarrhea caused by radiation therapy in patients with cancer. This is optimistic future research that could provide the basis of hope concerning the future of medical treatments [53].

### Tumor Microenvironment and Its Interation with Bacterial Therapies

The tumor microenvironment (TME) encompasses all elements surrounding a tumor, including various cell types and components such as immune cells, cancer-associated fibroblasts, blood vessels, extracellular matrix, and cytokines. Enduring complexion with bacterial therapies and cancer machineries undermines the significance of TME in modulating the efficiency of anti-cancer therapies. These interactions may further modify immune reactions, hypoxia, as well as nutrient access in the tumor leading to improved therapeutics [48].

**Immune modulation.** Bacterial therapies have been shown to enhance the immune responses through stimulating various immune cells, including T cells and natural killer cells, which are crucial for targeting and eliminating of tumor cells. This immunogenic activation promotes treatment with immunotherapies and other therapies. On the other hand, certain bacteria can inhibit certain immune activities; this is because tumors require immunity to be unnoticed by the immune system [46].

**Hypoxia.** Tumors often possess hypoxic areas with low concentrations of oxygen. Some bacteria can thrive in these low oxygen conditions and be engineered to target these areas specially. This capability allows delivering therapeutic agents selectively to hypoxic tumor sites, thereby reducing damage to surrounding healthy tissue [49].

**Nutrient availability.** Bacteria can also impact nutrients concentrations within the tumor microenvironment. They may compete tumor cells for essential nutrients or produce metabolites that affect the metabolism of the tumor cells. Some of these effects suppress tumor development and improve the ability of cancer cells to respond to other forms of therapy [45].

## Engineered Bacteria in Cancer Therapy

GEB is now used therapeutically more frequently for CRC and other diseases [54]. These engineered bacteria are like mini-factories that reside in the body tissues and the intestines to produce proteins and various molecular compounds that have the potential to cure [55]. They can influence the immune system, cure diseases, and act as Player reagents. It is now possible to develop sophisticated treatment methods to eradicate numerous diseases and cancers in particular optimally [56]. GEBs can be given personally or intravenously for different diseases, they modulate immune cells, suppress pathogenic bacteria, and cytotoxicity with foreign gene expression on the surface of tumor cells. Different GEBs are defined for some specialities for treating diseases, protecting the environment, and treating food industry applications. Some types of bacteria used in producing GEBs are *Salmonella*, Lactobacillus, and *Escherichia coli* are widely used in scientific research and health facilities [57].

### Bacteriophage Therapy

The growth rate of each cancerous growth is on the rise globally, and this pressure promotes the creation of efficient, effective, and cutting-edge treatment methods [58]. Regarding CRC treatment, bacteriophage therapy is a novel and relatively affordable approach that can inspire hope about the disease’s accessibility.The NF-κB pathway is associated with pro-inflammatory and pro-survival and it is enhanced in various cancer cells for tumor promotion and survival against cell death. Certain bacteriophages can inhibit the NF-κB signaling pathway by preventing the IκBα degradation. Such inhibition eliminates NF-κB from entering the nucleus and inhibits the expression of anti-apoptotic genes namely Bcl-2 and survivin leading to apoptosis in cancer cells. The PI3K/AKT signaling pathway, which regulates cell survival and cell proliferation is frequently dysregulated in CRC. Bacterial therapies can suppress this pathway by down regulating the phosphorylation of the molecule PI3K, resulting in low levels of the phosphorylated molecule AKT. This reduction diminishes downstream survival signals, thereby increase the apoptotic effect in cancer cells [60].

It is well-established that apoptosis, or programmed cell death is one of the vital processes that need to be activated in cancer therapy for the destruction of tumor cells. Microbial molecules can affect cytokine production including TNF-α that may either enhance or suppress pathways including NF-κB. NF-κB down regulation brings about a decrease in the levels of anti-apoptotic proteins such as Bcl-2 and so promote apoptosis in the cancer cells. Furthermore, p53 is a protein required by the organism to suppress tumor formation, and it is responsible for the cell’s decision to undergo apoptosis in the presence of DNA damage. It helps to regulate the pro-apoptotic genes and on the other hand, it suppresses anti-apoptotic pathways, whereas, it boosts up the apoptosis in CRC cells.[59]. In addition, some of the pathogenic bacteria and microorganisms have also been involved in CRC. Bacteriotherapy approaches have shown effectiveness in treating CRC with comparable or fewer side effects than typical illness medications (Table 1) [60].

**Bacteriotherapy mechanisms for treating CRC.** Bacteriotherapy is a potential strategy for CRC treatment based on applying bacterial characteristics for managing the disease. According to scientists, several significant modes of how bacteria act are known, for example, the formation of membrane pores, metastasis inhibition, tumor necrosis, and apoptosis [61]. Among the standard cell processes, apoptosis is essential in ker than in cancer treatment as it is a type of programmed cell death that affects the equilibrium of cell division and cell dying [62]. Two primary pathways govern apoptosis: two types, the intrinsic or mitochondrial pathway and the extrinsic or receptor-dependent pathway [63]. The intrinsic pathway works harmoniously with the necessary proteins, Bcl-1, Brx/Bcl-2-associated X protein, Bcl-2 adversary of Passing horrible cells, and BCL2 Executor/Bad guy 1/Bak [64]. Staggered, these proteins enable the leakage of the cytochrome c from the mitochondrion to the cytosol, leading to the formation of Caspase-9-bearing apoptosome complex followed by cell death. On the other hand, the extrinsic pathway is activated on ligand binding to the TNF-R receptor, recruiting Caspase-8 and then activating downstream caspase for apoptosis [65]. Nevertheless, these pathways communicate inextricably because the metabolites participating belong to one pathway may affect the other [66].

Thus, interference with these mechanisms in the bacteria mentioned eases the chances of developing efficient treatments for CRC. At some point, this microbial agent may provide a massive chance of formulating an ample treatment for bacterial illnesses, including CRC [67]. The conditions require that the compound be toxic to pathogenic cells to the highest extent, non-toxic to healthy cells, and more toxic to carcinoma cells. The subsequent section will discuss numerous techniques used in bacterial therapy for cancer, especially CRC-associated uses [68].

### Bacterial Colorectal Cancer Immunotherapy

The Immune system can fight against cancer and protect the body against tumor cells [69]. The immune system is bifunctional: it defends actively and repairs harm. It also creates immune cells to fight against cancer cells in defensive mode (Table 1) [70]. It calls for immunosuppressive cytokines and growth factors that support tissue remodeling and tumorigenesis in reparative mode. Bacteria may contribute to immune system mobilization in two ways: first, as pathogenic organisms; second, as colonizers of the host organism's body, as outlined in the subsequent sections [71].

**Activation of anti-tumor T-cell responses.** Lymphocyte response is significant in controlling cancer overgrowth since it can be regarded as an effector reaction. Regarding this, the anaerobic bacterium strain *E. coli* has a connecting influence with colorectal cancer (CRC) since it takes part in the host's defense mechanisms by getting the growth of the lymphocyte [72]. In CRC mouse models, these lymphocytes effectively eradicate the tumor, mainly comprising CD8+ T cells and CD4+ T cells. CD 8^+^ T cells are the primary effectors that clear cancers at this tumor recognition and destruction phase. On the other hand, CD8^+^ and CD4^+^ T cells are in the memory phase, which involves long-term immune surveillance [73].

Besides, CD8+ T cells can eliminate CRC, and CD4^+^ and CD8^+^ T cells were shown to kill newly formed tumors [74]. Also, regulatory T cells, CD4 + and CD25 + lymphocytes, have been used to note a decrease in colon disease. These cells can stimulate microbiota-associated immunotherapies, thus offering a new method of addressing colorectal cancer [75].

### Bacterial Biofilm Cancer Therapy

Biofilms are an early form of complex bacteria with large capacities of individuals affixed to a surface or protected by an extracellular matrix [76]. These structures shield bacteria from antimicrobial agents and host defense factors [77]. Biofilm formation is always connected with infections and contamination by *Salmonella* typhimurium, which can increase white blood cells [78]. Leucocytes are valuable for biofilm development, as ascertained by Rodis and companions (2020). Even though biofilms can be pathogenic to different diseases [79], it must be mentioned about the possibility of their application as a treatment method for some pathological processes [80].

Moreover, other related research has shown that bacteria biofilm formation can inhibit metastasis. By the time the SOS response is triggered on biopsy, these biofilms MIGHT be capable of killing cancerous cells [81]. The results of the present work suggest that bacterial biofilms can modulate metabolic rates and cell division of cancer cells. Therefore, bacterial biofilms are considered a promising cancer treatment option [82]. Furthermore, the studies prove that the bacterial proteins and DNA are required in biofilm building and stop the dissemination of the cancer cells [83]. For example, *S. agalactiae* synthesizes polysaccharides that prevent cancerous stem cells from adhering to endothelial cells, hindering one of the vital steps in spreading infection and metastasis [84, 85].

### Bacterial Anticancer Metabolites

The other therapeutic strategy in treating CRC can be advised from bacterial peptides as anticancer agents. Some researchers also confirmed that bacteria use these peptide toxins to inhibit tumor growth [86]. Bacteriocins are one of the subtypes of bacterial peptides - proteins that, under optimal conditions of the host organism's physiological environment, act on target cells, causing their lethal outcome, being produced by microorganisms as weapons against competitors [87, 88] (Table 1). Two categories of bacterial anticancer toxins: first, toxins that attach themselves to the surface antigens on malignant cells, and second, toxins that attach themselves to the ligands on cancer cells. CRC cells have a lot of growth-specific antigens on their outermost layer, most of which function as receptors; bacterial toxins are a beneficial way to link them [89]. The upcoming segment will show a subset of a few bacterial toxins vehemently opposed to CRC therapy [90].

### A. Enterotoxin

For treating colorectal cancer, the gram-positive, anaerobic spore-forming bacterium *C. perfringens* produces the enterotoxin CPE [91]. These toxins aid in the treatment by disrupting the osmotic balance of cells and causing cancer cell death by binding to the Claudin-3 and -4 surface receptors, which are abundantly present in cancer cells. Pahle *et al*. also discovered the perfect vector for CPE expression, which targets Claudin-3 and Claudin-4 in colon cancer cells (Caco-2, HT-29, SW480, HCT-116, and SW620) [92]. The CPE-based gene transfer method targets Claudin-3 and Claudin-4, effectively killing tumor cells and offering a promising approach for colorectal cancer (CRC) treatment [93].

### B. Colicin

Members of the Enterobacteriaceae family release bacteriocins, including colicins, that show cytotoxic effects on numerous tumor cells [94]. The mode of action of colicins includes cytotoxic effects, which include the formation of pores, DNase and RNase activity, and murein synthesis inhibition [95, 96]. Studies have also followed the effects of colicin E1, E3, A, and U in the reduction of growth of HT-29 colon adenocarcinoma and other human cancer lines. Therefore, it can be concluded that Colicin E1 was not cytotoxic to HT 29 cells, but the highest toxicity was recorded in the case of Colicin A [97, 98].

### C. Microcin

Microcin E492 is produced by the Gram-negative bacterium: Klebsiella pneumonia secretion indicates high cytotoxicity in different tumor cells such as HeLa and Jurkat cells [99]. Thus, Microcin E492 induces apoptosis by forming pores in the cell membrane and inhibiting TLR4 signaling [100]. For instance, microcin E492 is toxic solely to cancer cells but has little or no effect on bone marrow cells, splenocytes, KG-1 cells, and human tonsil cells [101]. This selective cytotoxicity makes microcin E492 a good candidate for creating an effective anticancer drug [102].

### Bacterial Cancer Therapeutic Carriers

*Mechercharimyces* spp. Synthesize the cyclic thiopeptide urukthapelstatin A, which has potent cytotoxic activity against colorectal cancer cells [103, 104]. This compound has potential for treating colorectal cancer that targets metastases by microbial transporters [105]. Furthermore, imaging techniques involving bioluminescence have allowed for the visualization of colonization and metastases of diseases without invasiveness; such advances present new possibilities for detecting and treating the disease in the future. This strategy enables the identification and staging of tumors in the body, such as colorectal cancer (CRC) and metastases [106, 107].

*Listeria monocytogenes* is a cancer immunotherapy vector that increases the strength of anti-tumor activities through CD8^+^ and CD^4+^ lymphocytes, crossing epithelial living layers and stimulating the immune system. New bacterial strains like Clostridium novyi-NT have become eligible therapies [108]. The C. novyi-NT, a non-pathogenic strain, grows in mouse models and targets only CRC cells in internal areas, thereby eliminating them. Clinical results showed that the C. novyi-NT strain and conventional chemotherapy caused hemorrhagic necrosis within 24 h of administration and were possibly effective in weakening CRC cells [109]. As a result, using microbes in terms of vectors is indispensable for defining, influencing, and eliminating threatening cells and metastatic formations in the given kind of oncology - colorectal cancer treatment [110].

### Bacteria-Mediated Anti-Angiogenesis Therapy

Angiogenesis therapy is one of the new cancer treatment methods where bacteria are used to prevent the growth of blood vessels that are ubiquitously necessary for tumor growth and metastasis. Thus, in terms of the anti-angiogenic approach, the efficacy of this strategy to decelerate or halt cancer growth is promising [111]. A recent study at poC_005 used genetically attenuated *Salmonella* sp. and rhEndostatin, an angiogenesis inhibitor. Isolated bacterial strain (VNP20009) to specifically inhibit angiogenesis on syngeneic tumor-bearing mice. This synergistic approach greatly suppressed tumor growth while monotherapy barely affected growth duplication [112, 113]. This is an implication that the occurring metabolism can enhance or even augment the angiogenesis-provoking impact of therapeutic agents [114]. Using endostatin vectors *Bifidobacterium longum* (Table 1) [114, 115] and B. teenagers is suggested, which can directly aim angiogenesis for stopping the cancer tropism. Niethammer *et al*. evidenced that an oral anti-angiogenic bacterial DNA vaccination of VEGFR-2 using mouth gauze, given by a weak form of *Salmonella* typhimurium, substantially constrained the growth of numerous cancer cells, especially the colon cancer [116, 117].

## Molecular Genetic Engineering Approaches

Despite advances in conventional colorectal cancer (CRC) treatments, like surgery, chemotherapy, and radiation therapy, a significant proportion of patients remain refractory to therapy, particularly in advanced stages. Molecular genetic engineering is a relatively new and potential field for designing new and effective CRC treatment strategies [118]. From the continuously developing understanding of the molecular basis of CRC, genetic engineering means that molecular genetic techniques bid to create a particular and individualistic approach to a patient's care [119, 120]. These strategies include scientifically altering cancer cells' genes to rectify or inhibit the improper signals or genes that cause cellular malignancy and invasion. Some include Gene correction, Virus-directed enzyme prodrug therapy, Immunogenetic therapy, and Oncolytic virotherapy [121].

The development and the spread of CRC depend on multiple genetics and epigenetic changes. The increased number of molecularly targeted therapies and the studies based on the mechanistic investigation of essential targets are considered to be a breakthrough in the treatment of CRCs. RBPs and RNA work hand in hand to modulate gene regulation events, including RNA processing, splicing, localization, and translation. Since tumor cells are dependent on RBPs because of the intensive RNA synthesis, the latter can be considered potential targets for CRC treatment [122]. The latest techniques of gene-editing such as CRISPR-Cas9 are enhancing bacterial therapies for colon cancer because it is altering bacterial DNA accurately. CRISPR-Cas9 can be used to program bacteria to specifically recognize cancer cells, modify bacterial metabolites to exert anti-tumor effects or to stimulate immune system’s response [123]. This precision allows minimizing damage to nearby healthy tissue and cells by programming bacteria only to function in the tumour environment. Also, newer techniques such as the prime editing and other gene silencing techniques give even better control in the genetic modifications, thus leading to the creation of highly specific bacteria strains such as *E. coli* and *Salmonella* typhimurium that could efficiently combat colorectal cancer. These innovations offer an opportunity for developing individual and targeted therapies for cancer based on molecular profile of tumors [124].

### CRISPR-Cas9

CRISPR-Cas9 has become one of the most powerful tools for genome editing since it allows for the alteration of specific nucleotides in various organisms. In case of colorectal cancer, a new avenue of attack for cancer has been made possible by CRISPR-Cas9 which targets specific genes that are involved in the progression of cancer. However, the use of CRISPR-Cas9 comes with a significant challenge, which is off-target effect, whereby the Cas9 enzyme cleaves other regions in the genome than the target region. These off-target can cause unwanted insertions, deletions, or rearrangements of DNA sequence. This can lead to the occurrence of increased genetic variations that are unfavourable in cancer treatments [125]. Furthermore, the used drug might produce off-target effects by interfering with oncogenes or tumor suppressor genes that were not intended to be affected. This can undermine the goals of cancer treatment, as it will promote rather than suppress it. Additionally, off-target activity allows undesired gene disruption; thus, resulting in cell death or dysfunction, when necessary, genes get disrupted and yield undesirable side effects when other vital mobile activities are interfered with. In colorectal cancer, a disease type with rather high mutation rate and chromosomal instability, off target effects represent a high risk that can threaten effective application of CRISPR based therapies on safety grounds [126]. Minimizing such side effects is important in order to improve the efficiency and safety of gene modification in cancer therapy.

**Mechanism of CRISPER.** CRISPR-Cas9 gene editing process is as follows: Firstly, particular guide RNA or gRNA is specifically designed by the researchers or selected by them containing the sequence in complementary with the targeted DNA sequence. Subsequently, the gRNA is transduced into the target cell, with the Cas9 enzyme forming the gRNA-Cas9 complex [127]. It then follows and anneals the target DNA sequence so the gRNA can engage the target segment and correctly position the Cas9 nuclease for its incisions. It must be noted that the Cas9 enzyme cuts the DNA at the target site on the given DNA molecule, creating a double-strand break on the DNA molecule [128].

**Fig. 4:**
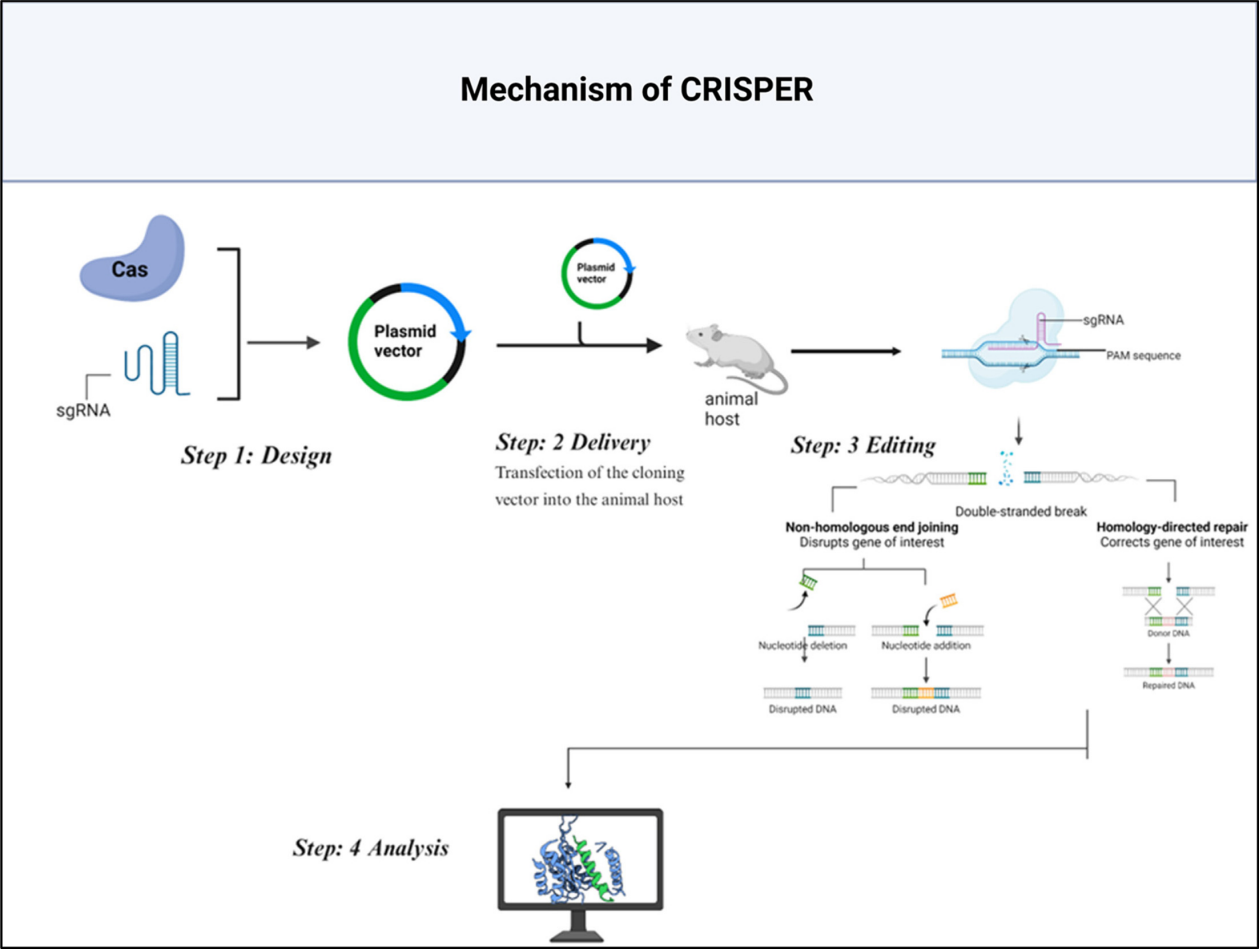
CRISPR-Cas9 mechanism: A step-by-step guide to genome editing [126].

After that, this double-strand break triggers the cell's repair machinery, activating two primary processes: There are two main pathways for DNA double-strand or repair: non-homologous end joining (NHEJ) and homologous recombination (HR). Another DNA repair mechanism is non-homologous end joining (NHEJ), which is faster than the other DNA repair mechanisms. It is fast but can lead to a frame-shift mutation with small insertions or deletions (indels) at the cut site and may completely knock out the gene. On the other hand, HR, or the “conventional repair method,” relies on a format by the researchers to repair the break accurately so that only the gene of particular interest is edited. CRISPR-Cas9 system can be used in different techniques to change genes, such as cutting the genes, replacing one gene with another, or even silencing genes temporarily without causing structural changes in the DNA segment (Fig. 4) [129].

**Recent advancements in reducing off-target effects.** To enhance the safety profile of CRISPR-Cas9 for therapeutic use, especially in cancer treatment, several strategies and technological advancements have been developed to minimize off-target effects [125]:

***High-fidelity Cas9 variants:***

*SpCas9-HF1.* This Cas9 variant was engineered to have additional changes on the same protein that helps to reduce the off-target effects through decrease in non-specific binding to DNA, whilst the on-target effects and efficiency is not compromised [130].

*eSpCas9*. Enhanced specificity Cas9 optimizes on the Cas9 protein and does not allow it to interact with the DNA phosphate backbone hence causing minimal off-targeting.

*HypaCas9.* This Cas9 variant includes mutations which make the enzyme more potent by raising the stringency of PAM detection by the enzyme and reducing off-target activity.

***Base editing technologies*:**

*CRISPR base editors*. These editors allow for targeted gene modification: Increase the frequency of targeted single nucleotide substitutions without creating double strand breaks and, therefore, the risk of off-target mutations.

*Prime editing.*A more recent method of CRISPR that makes it possible to integrate, delete and modify DNA without the formation of double strand breaks, avoiding the typical error prone repair mechanisms associated with traditional CRISPR methods.

***Guide RNA optimization:***

*Truncated guide RNAs (tru-gRNAs)*. Extending the history of gc notch guide RNA design, reducing the length of guide RNA to enhance specificity through decreasing off-target effects.

*Chemical modifications*. The improvement of guide RNA stability and the minimization of off-target effects can be achieved using chemically modified nucleotides, for instance 2’-*O*-methyl and phosphorothioate linkage [131].

***Dual Cas9 nickases:***

Introducing two Cas9 nickases to show single-strand break instead of double-strand breaks that have higher off-target activity because only two nickases are required to be positioned tightly to one another to make double-strand cuts [127].

**Application of CRISPR-Cas9 in cancer.** Lately, genome editing technology, CRISPR-Cas9, has been used effectively for deducing the CRC tumorigenesis and explor the order of mutations leading to CRC, a procedure to trace CRC development and study the natural history of CRC [130]. The options of target genes and the possibility of modifying their presence in the genome give CRISPR-Cas9 high efficiency in its work - it can add and delete genes. It has been found using a genome-specific CRISPR-Cas9 knockout screen that specific metabolisms are affected only when KRAS is active on cells, which may have future therapeutic benefits for cancer metabolism in a patient's mutant KRAS tumor, exhibiting some therapeutic application [131].

To investigate the functions of β-catenin, the researchers repaired the mutation in the HCT-116 human colon carcinoma-derived cell line using CRISPR-Cas9. This restoration of Wnt phosphorylation reduced the rate of β-catenin getting translocated into the nuclear compartment and caused the downregulation of survivin and c-myc. Therefore, cell proliferation was suppressed and the tumors’ capability to form in the mouse xenograft models was impacted negatively [132]. Moreover, intervened four genes with a high mutation rate and efficacy into human intestinal stem cells and obtained tumors translating adenocarcinoma-like histological features while understanding the critical driver mutations [133].

Further studies proved the applicability of the CRISPR-Cas9 application for genome editing in living organisms and for transplanting colon tumors to mice that did not have such mutations [134]. Besides, for the further identification of driver mutations, CRISPR-Cas9 has consistently been used to target other genes, including Acvr2 a, Acvr1 b, and Arid2, or to study the MUC5AC-CD44 signaling pathway based on which more evidence has been provided concerning the genetic features of CRC [135].

### Prime Editing

On the molecular level, prime editing is a new gene editing system based on the CRISPR-Cas 9 [136]. Dr. David Liu and his team at the Broad Institute of MIT and Harvard discovered that prime editing is versatile and efficient compared to CRISPR-Cas9 for modifying DNA sequences. Prime editing does not cause double-strand breaks in DNA strands, as in the case of CRISPR-Cas9. Instead, it utilizes a designed RNA molecule, the prime editor, to enable the incorporation, exclusion, or exchange of a particular DNA sequence. The prime editing process involves three main components: One of them is a prime editing guide RNA (pegRNA), the second is Cas9 nickase enzyme, and the third one is reverse transcriptase. The first strand of the target DNA is cut when the peg RNA takes Cas9 nickase to the target site on the DNA. The reverse transcriptase has to then utilize the pegRNA as a template to form a new DNA strand with the required changes. Lastly, the ionic forces of the cell's DNA repair equipment seal the deal by replacing the faulty DNA strand [137].

Comparing prime editing with CRISPR-Cas9 genome editing, the former has these advantages: point mutation insertions and deletion, with no requirement of double-strand breaks and the problems connected to the same [138]. Moreover, prime editing allows for integrating brand-new DNA segments in the desired genome region compared to base editing and predication editing. While prime editing is a relatively new method, favorable outcomes have been reported, with edits made in human cells such as genetic disorders such as sickle cell disease and Tay-Sacks disease. The technique of prime editing has also been utilized in plants and animals to better serve agriculture and biomedical purposes [139].

### Gene Silencing

The process starts with forming dsRNA that can enter the cell and, with the help of a Dicer enzyme, is cleaved into small interfering RNA (siRNA) of 21-23 nucleotides. Subsequently, the RNA-activated silencing complex (RISC) compels these small parts with the single-stranded RNA, or antisense siRNA, to match the target mRNA. This binding prevents gene expression and represses or knocks down the target genes. Additionally, the siRNA antisense strand can initiate the formation of dsRNA with the help of RNA-dependent RNA polymerase (RdRp), leading to the breakdown of mRNA through RNAi cycling (Table 2) [140].

RNAi has high specificity and efficiency, allowing for the simultaneous inhibition of multiple genes in the same signal pathway. Furthermore, the transfer of RNA from one cell to another can impact the expression of subsequent gene generations. However, issues with toxicity, off-target effects, efficacy, and efficient in vivo delivery systems remain. Despite these challenges, clinical trials are underway for over ten distinct RNAi-based cancer treatments, targeting genes that contribute to disease progression, metastasis, cell death, mitosis, and signaling pathways [141].

### Suicide Gene Therapy

Suicide genes are a promising approach in cancer therapy, leveraging three primary mechanisms to eradicate tumor cells. Firstly, the direct insertion of a suicide gene into tumor cells triggers a cytotoxic effect, converting non-toxic prodrugs into toxic substances that disrupt DNA synthesis and induce cell death. Secondly, viral vectors like Lenti-LEP503-HSV-tk/GCV, combined with the bystander effect of ATRA, can effectively target and kill tumor cells. This strategy enhances the killing effect by exploiting the bystander effect [142].

Thirdly, suicide gene products are capable of being controlled through changes in some signaling pathways, such as Wnt and Notch, to induce apoptosis or necrosis in the cancer cells. Through the promoters of genes such as survivin, hTERT, AFP, Cox2, E2F1, and FOS, it is possible to target with precision the suicide genes. The human telomerase reverse transcriptase associated with cell cycling and cancers is hTERT. hTERT gene codes for the telomerase enzyme synthesis, which significantly protects the DNA helix. Limiting hTERT levels indicates improvements to the treatment as well as potential biomarkers that have already been deemed of valuable use in characterizing the prognosis of colorectal cancer; cellular senescence has also been discovered to work as a means of enforcing treatments on colorectal cancer [143].[Table T3]

## Discussion and Perspectives

However, there is little appreciation for the therapeutic uses of the little organisms capable of eradicating unpleasant diseases. Nevertheless, according to researchers, there are bacterial species that can promote patients' recovery but, at the same time, contribute to malignant processes. However, several strains can adversely affect human tissues [144]. Colorectal cancer (CRC) develops and progresses through three different pathways: These include chromosomal instability (CIN), microsatellite instability (MSI), and CpG island methylator phenotype (CIMP). This knowledge about these molecular pathways is essential in enhancing the diagnosis and management of CRC for enhanced therapeutic outcomes.

Some applied microorganisms can be potential drug carriers because they have sizes below 0.2 biting of sub-nuclear and non-water resistant. This treatment shows fewer side effects than other cancer therapies and can employ bacterial specialists or their enzymes to treat CRC. Furthermore, microorganisms not associated with the extracellular and intracellular receptors can use bacterial peptides to target numerous blockage processes [145]. However, bacterial peptides' half-life is shorter, which presents an application-related challenge; more effort must be put into addressing this issue.

The complexity of microorganisms and the microbiome and their relationship hold significant implications for essential applications. The activation of anti-tumor T-cell responses through bacteria-mediated approaches further highlights the potential of immunotherapy. Bacterial vectors effectively present tumor antigens, generating a robust and specific immune response. Ongoing clinical trials are investigating this method, which has demonstrated promise in preclinical models. Furthermore, certain bacteria produce metabolites with potent anticancer properties, including apoptosis induction, cell proliferation inhibition, and angiogenesis disruption in cancer cells [142].

Although bacteriotherapy shows promise, it may not entirely address the healing process; microorganisms as a creative, restorative methodology in colorectal disease are highly anticipated from various perspectives. Harnessing the unique microbiome of each patient to tailor treatment, combined with bacteriotherapy potential for disease prevention or harmful agent carrier therapy, could enhance microbe-mediated action against colorectal cancer. However, further in vivo tests, fundamental clinical trials, and clear evidence from microbiota-based perspectives are necessary to establish bacteriotherapy as a significant treatment method for colorectal cancer [146].

Recent advancements in molecular genetic engineering, particularly the CRISPR-Cas9 system, offer precise genome editing capabilities for CRC treatment. The CRISPR-Cas9 system effectively removes oncogenes, repairs tumor-suppressor genes, or introduces therapeutic genes [147]. This technology's precise editing capability has significant implications for targeted cancer therapy, enabling researchers to develop innovative treatment strategies. Integrating Man + AI can enhance the quality of treatment strategies used in different research, enhancing the quality of results from patient treatment. This approach will foster the identification of specific solutions to patients' unique characteristics and positively impact the treatment of CRC (Dalal *et al*., 2020).

## Conclusion

In conclusion, bacteriophage administration combined with new genetic engineering approaches is a breakthrough in colorectal cancer treatment. Applying microorganisms' best qualities and using genetic editors, such as CRISPR-Cas9 and prime editing, opens up the opportunity to create practical and particular but less toxic treatments that change the catastrophic destiny of the sick. Additional studies and intervention trials are necessary for this model's full benefits. The prospects of bacteriotherapy - significant findings: It is with great enthusiasm that such data outline the extensive potential of bacteriotherapy in managing colorectal cancer. Further research on the interaction of the microbiome, the immune system, and cancer cells can reveal even more efficient approaches to prevent and treat the disease, resulting in better patients' quality of life and lives saved.

## Figures and Tables

**Table 1:** Engineered bacteria and their therapeutic approaches in colorectal cancer (CRC).

Section	Description	References
Bacteriophage Therapy	Bacteriophages are viral agents that target bacteria precisely, making them a promising tool in the fight against colorectal cancer. Compared to conventional drugs, they can be effective with fewer side effects.	[59, 60]
Bacterial colorectal cancer immunotherapy	This innovative approach harnesses the ability of *Salmonella* typhimurium and *Escherichia coli* to activate inflammasome pathways, triggering anti-tumor T-cell response and cytokine-mediated tumor necrosis. This strategy enhances antitumour immunity by exploiting the symbiotic relationship between bacteria and the host immune system, offering a promising avenue for effective CRC therapy.	[70]
Bacterial biofilm cancer therapy	Bacterial biofilms exert anti-metastatic effects by inhibiting cancer cell dissemination and modulating metabolite profiles, thereby constraining malignant progression.	[78, 79]
Bacterial anticancer metabolites	Bacterial peptides and toxins produced by species such as *Clostridium perfringens*, *K. pneumoniae*, and *Pediococcus acidilactici* exhibit potent cytotoxicity against colorectal cancer cells through targeted mechanisms involving enterotoxins, colicins, microcins, and pediocins.	[98]
Bacterial cancer therapeutic carriers	Bacteria such as *Listeria monocytogenes* and C. novyi-NT are being repurposed as vectors for targeted delivery of therapeutic agents and imaging modalities, enabling precise localization and visualization of colorectal cancer.	[104]
Bacteria-Mediated Anti-Angiogenesis therapy	Bacteria such as *Salmonella* sp. and *Bifidobacterium longum* inhibit angiogenesis, stifling tumor growth by cutting off its vascular supply. This targeted approach shows promise as a synergistic component of anticancer therapies, enhancing treatment efficacy.	[114]

**Table 2:** Additional considerations in the CRISPR-Cas9 gene editing process are as follows.

Consideration	Description	References
Off-target effects	Occasionally, CRISPR-Cas9 may unintentionally cut non-targeted sites, leading to off-target effects.	[125]
Mosaicism	CRISPR-Cas9 can create mosaicism, where only some cells in a population are edited, resulting in a mixture of edited and unedited cells.	[127]
Gene regulation	In addition to gene editing, CRISPR-Cas9 can regulate gene expression, allowing for temporary or reversible control of gene activity.	[126]

**Table 3 T3:** Comparison of molecular genetic engineering techniques.

Genetic Engineering Approach	Mechanisms	Key components	Precision	Impact on CRC therapy	Advantages	References
CRISPR-Cas9	Creates double-strand breaks in DNA for targeted modification	Guide RNA, Cas9 enzyme	High	Gene correction, functional gene screening, targeting specific mutations (*e.g.*, KRAS)	Development of a safer delivery system, expansion of clinical applications	[131]
Prime editing	Uses a prime editor for targeted DNA sequence changes without double-strand breaks	Prime editing guide RNA (pegRNA), Cas9 nickase, reverse transcriptase	Very high	Precision editing of genetic mutations, the potential for correcting diseases like sickle cell and Tay-Sachs	Further optimization of efficiency	[137,138]
Gene Silencing	Uses siRNAs to inhibit mRNA expression, leading to gene silencing	siRNAs, RNA-induced silencing complex (RISC)	High	Targeting gene expression in CRC progression, metastasis, and signaling pathways	Integration with other therapies, overcoming delivery and stability challenges	[140]
Suicide Gene Therapy	Introduces suicide genes that convert prodrugs into toxic agents or manipulate pathways to induce cell death	Suicide genes, viral vectors, prodrugs	Moderate	Direct tumor cell killing, leveraging bystander effects, and manipulating pathways like Wnt and Notch	Exploring new suicide gene constructs, enhancing specificity, and reducing side effects	[142]
